# DNA methylation is differentially associated with glycemic outcomes by different types of weight-loss interventions: an epigenome-wide association study

**DOI:** 10.1186/s13148-023-01522-9

**Published:** 2023-07-01

**Authors:** Xiaoxiao Wen, Helena Palma-Gudiel, Guanhong Miao, Mingjing Chen, Zhiguang Huo, Hao Peng, Stephen Anton, Gang Hu, Ricky Brock, Phillip J. Brantley, Jinying Zhao

**Affiliations:** 1 Department of Epidemiology, College of Public Health and Health Professions and College of Medicine, University of Florida, 2004 Mowry Road, CTRB 4230, Gainesville, FL 32610 USA; 2 Department of Biostatistics, College of Public Health and Health Professions and College of Medicine, University of Florida, Gainesville, FL USA; 3 Department of Epidemiology, School of Public Health and Jiangsu Key Laboratory of Preventive and Translational Medicine for Geriatric Diseases, Medical College of Soochow University, Suzhou, China; 4 Department of Aging and Geriatric Research, University of Florida, Gainesville, FL USA; 5 Chronic Disease Epidemiology Laboratory, Pennington Biomedical Research Center, Louisiana State University System, Baton Rouge, LA USA; 6 Behavioral Medicine Laboratory, Pennington Biomedical Research Center, Louisiana State University System, Baton Rouge, LA USA

**Keywords:** DNA methylation, Epigenetics, Obesity, Bariatric surgery, Weight loss, Epigenome-wide association study

## Abstract

**Background:**

Alterations in DNA methylation (DNAm) have been reported to be a mechanism by which bariatric surgeries resulted in considerable metabolic improvements. Previous studies have mostly focused on change in DNAm following weight-loss interventions, yet whether DNAm prior to intervention can explain the variability in glycemic outcomes has not been investigated. Here, we aim to examine whether baseline DNAm is differentially associated with glycemic outcomes induced by different types of weight-loss interventions.

**Methods:**

Participants were 75 adults with severe obesity who underwent non-surgical intensive medical intervention (IMI), adjustable gastric band (BAND) or Roux-en-Y gastric bypass (RYGB) (*n* = 25 each). Changes in fasting plasma glucose (FPG) and glycated hemoglobin (HbA1c) were measured at 1-year after intervention. DNAm was quantified by Illumina 450 K arrays in baseline peripheral blood DNA. Epigenome-wide association studies were performed to identify CpG probes that modify the effects of different weight-loss interventions on glycemic outcomes, i.e., changes in FPG and HbA1c, by including an interaction term between types of intervention and DNAm. Models were adjusted for weight loss and baseline clinical factors.

**Results:**

Baseline DNAm levels at 3216 and 117 CpGs were differentially associated with changes in FPG and HbA1c, respectively, when comparing RYGB versus IMI. Of these, 79 CpGs were significant for both FPG and HbA1c. The identified genes are enriched in adaptive thermogenesis, temperature homeostasis and regulation of cell population proliferation. Additionally, DNAm at 6 CpGs was differentially associated with changes in HbA1c when comparing RYGB versus BAND.

**Conclusions:**

Baseline DNAm is differentially associated with glycemic outcomes in response to different types of weight-loss interventions, independent of weight loss and other clinical factors. Such findings provided initial evidence that baseline DNAm levels may serve as potential biomarkers predictive of differential glycemic outcomes in response to different types of weight-loss interventions.

**Supplementary Information:**

The online version contains supplementary material available at 10.1186/s13148-023-01522-9.

## Background

Severe obesity, defined by a body mass index (BMI) of ≥ 40 kg/m^2^ or ≥ 35 kg/m^2^ with comorbidities (e.g., diabetes), is a serious health condition that develops from the complex interplay between genetic and environmental factors [1]. In addition to profoundly affecting quality of life, severe obesity substantially increases total and cause-specific mortality due to many major chronic diseases, such as coronary heart disease, type 2 diabetes (T2D) and cancer [2, 3]. While various treatment approaches have been developed, bariatric surgery has proven to be the most effective, superior to lifestyle modification or medication in terms of substantial and sustained weight control [4]. Among the common bariatric surgical procedures, Roux-en-Y gastric bypass (RYGB) has been reported to achieve a sustained and greater weight loss as well as a higher rate of T2D remission compared to adjustable gastric band (BAND) [5, 6]. Previous clinical studies have documented a large variability in weight reduction and glycemic control following RYGB or BAND [7, 8], indicating that specific patient groups may respond differentially to different surgical procedures.

Epigenetic regulation, particularly DNA methylation (DNAm), may provide a link between genetic and environmental factors in the development of complex diseases such as obesity and T2D [9]. Alterations in DNAm have been reported to be a mechanism by which bariatric surgeries resulted in considerable metabolic improvements in the patients [10–12]. Notably, there is suggestive evidence that maternal bariatric surgeries on mothers may also affect the metabolic health of their offspring through altered DNAm [12]. However, previous studies have mostly focused on change in DNAm following weight-loss interventions [10, 11, 13, 14], yet whether DNAm prior to intervention can explain postoperative glycemic outcomes has not been investigated. Patterns of DNAm resulting from obesity or other exposures may be differentially malleable to particular interventions, and thus may underlie the large variability in the glycemic outcomes observed. Identifying baseline DNAm patterns that modify the treatment effects of different types of interventions will enhance our understanding of the mechanism underlying the favorable outcomes of bariatric surgeries. In addition, it may have important implication for treatment decisions through the assessment of DNAm patterns that are linked to effective response to specific interventions. To this end, the goal of this study is to examine whether baseline peripheral blood DNAm is differentially associated with glycemic outcomes induced by different types of weight-loss interventions.

## Methods

### Study participants

The current analysis included 75 patients with obesity who participated in the HeadsUp Study (July 2011–June 2016, Louisiana, United States). Detailed information for the study design and methods of the HeadsUp study has been published previously [15]. Briefly, HeadsUp is a state-funded longitudinal study aiming to assess the effectiveness of different types of weight-loss interventions among over 1400 adults with severe obesity. Treatment options included a non-surgical intensive medical intervention (IMI) program, consisting of a combination of structured diet and behavioral management, as well as three different types of bariatric surgery: BAND, RYGB, and sleeve gastrectomy (SG). Eligible participants for the study were volunteers aged 21 to 70 years, with a BMI ≥ 33 kg/m^2^ if they were interested in IMI, and a BMI ≥ 40 kg/m^2^ or BMI ≥ 35 kg/m^2^ and T2D if interested in bariatric surgeries. Exclusion criteria included prior bariatric surgeries, significant medical conditions, psychiatric disorders, or inability to comply with the protocol. Pregnant women or women who intended to become pregnant within 3 years were also excluded.

We randomly selected 25 participants from each intervention arm (IMI, BAND and RYGB) for DNAm profiling using genomic DNA isolated from peripheral blood collected prior to intervention. Since SG was introduced to HeadsUp after the collection of genomic data, the current analysis did not include participants from the SG group. This study was approved and monitored by the Institutional Review Board at Pennington Biomedical Research Center. All participants provided written informed consent.

### Clinical measures

Demographic information, history of diabetes and use of hypoglycemic medications were collected at enrollment using standard questionnaires, as previously described [16]. Measurements for body weight, height, waist circumference, blood pressure, FPG and HbA1c were collected at baseline and 1-year after intervention. Weight, height, and waist circumference were measured using a standardized protocol in which participants wore light clothes and no shoes. BMI was calculated as the body weight in kilograms divided by the square of the height in meters. Blood pressure was measured by trained study staff according to a standard protocol adapted from the procedures recommended by the American Heart Association [17]. FPG and HbA1c were measured using blood samples obtained after an overnight fast. Approximately 60 mL of fasting blood was drawn into EDTA tubes by trained phlebotomists at the Pennington Biomedical Research Center Clinical Research Laboratory. Diabetes was defined as FPG ≥ 126 mg/dL, HbA1c ≥ 6.5%, or use of antidiabetic medications. The glycemic outcomes of interest in the present analyses included the changes in FPG and HbA1c measured after weight-loss intervention.

### Genome-wide DNA methylation analysis

Genome-wide DNAm levels were quantified by the Illumina Infinium HumanMethylation450 BeadChip using DNA isolated from peripheral blood leukocytes at baseline. Briefly, genomic DNA was isolated using the QIAamp DNA Mini kit (QIAGEN Nordic, Sollentuna, Sweden), followed by bisulfite treatment using the EZ-96 DNA methylation-Gold kit (Zymo Research, Irvine, CA, USA). The modified DNA was then used to measure DNAm according to manufacturer’s instructions (Illumina Inc., San Diego, CA). All samples were measured within the same plate. Pre-processing, quality control, and normalization of the DNAm data were performed using R package minfi [18]. Functional normalization was carried out using the preprocessFunnorm function of minfi. Samples were excluded if more than 1% of the probes had a detection *P* value > 0.01 or when predicted sex did not match reported sex. Probes were excluded if more than 10% of the samples had a detection *P* value > 0.01 (*n* = 560), located within known SNPs (dbSNP 137 Common database) in either the CpG site or at the single base extension site (*n* = 17,516), or on the sex chromosomes (*n* = 11,453). All 75 samples and 455,983 CpG sites passed quality control and were included. Methylation beta-values were used for the data analyses.

### Statistical analyses

Statistical analyses were performed using R version 4.1.1.

**Fig. 1:**
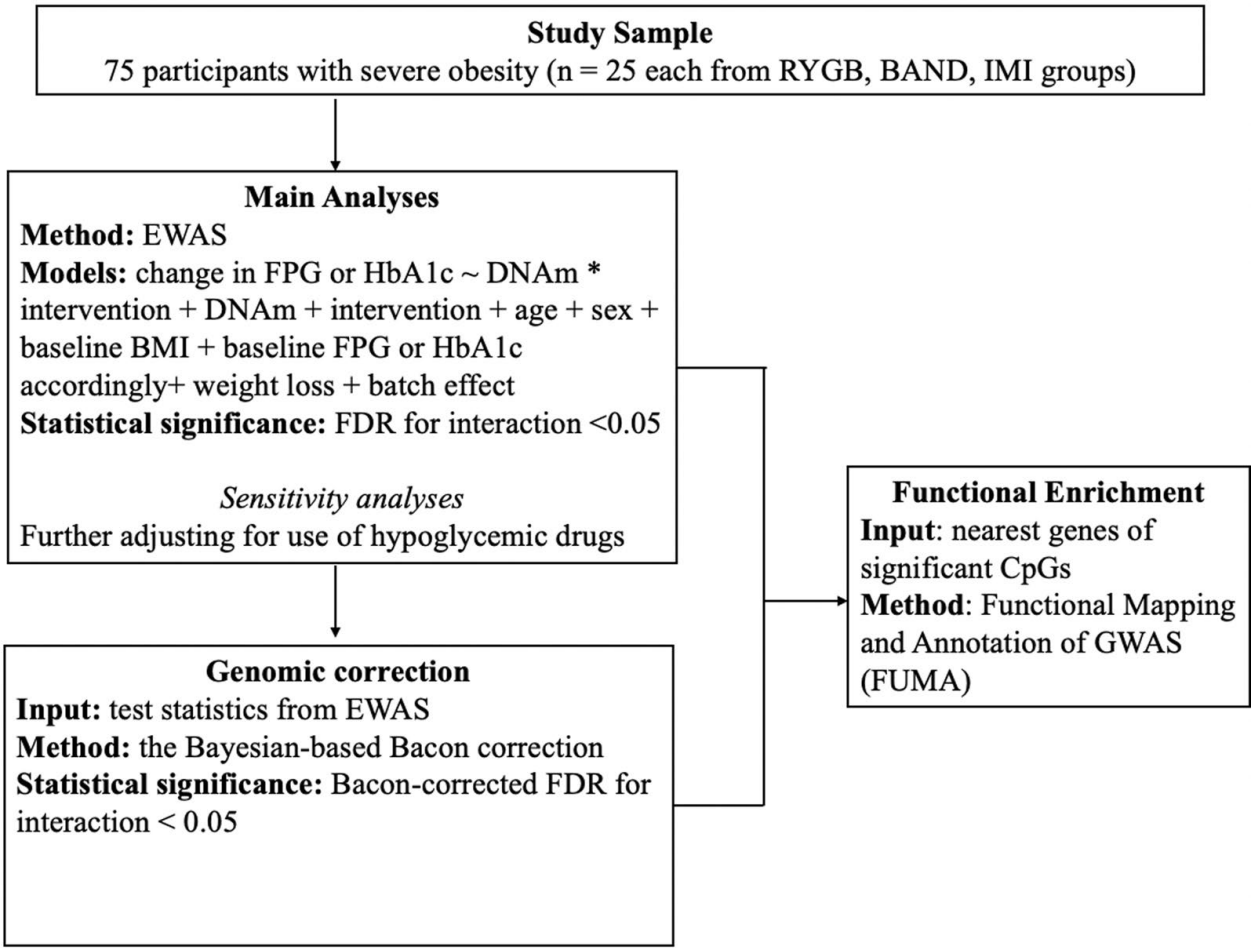
Analysis flowchart. Abbreviations: BAND, adjustable gastric band; EWAS, epigenome-wide association study; FDR, false discovery rate; FPG, fasting plasma glucose; HbA1c, hemoglobin A1c; IMI, intensive medical intervention; RYGB, Roux-en-Y gastric bypass

Clinical characteristics of study participants were summarized with continuous measures expressed as mean ± standard deviation (SD) or median with interquartile range as appropriate, and categorical measures as frequency with percentage. Difference in baseline characteristics among the three intervention groups was tested by one-way ANOVA, Chi-square test or Fisher’s exact test. Changes in clinical characteristics were calculated as the measurement at 1-year follow-up minus the baseline value and were tested by paired t-test or Wilcoxon rank test for statistical significance. Figure 1 is a flowchart describing the analytic plan of this study.

### Epigenome-wide association studies

Epigenome-wide association studies (EWAS) were performed using linear regression models to identify baseline methylated CpGs that were differentially associated with glycemic outcomes in response to different types of weight-loss interventions. Specifically, each glycemic outcome of interest (FPG or HbA1c) was modeled as the dependent variable, while intervention type (RYGB, BAND and IMI), DNAm, and their interaction were modeled as independent variables. We included an interaction term in the model as we were especially interested in testing whether DNAm modifies the different impacts of weight-loss interventions on post-operative glycemic outcomes. All models were adjusted for age, sex, baseline BMI, baseline value of the outcome variable and weight loss. Additionally, potential batch effects and other sources of variation were examined and adjusted with the R package SVA [19]. The identified surrogate variable highly correlated with cell type compositions estimated by minfi, and thus was also adjusted in the models to control for cell type compositions and other sources of undetected systematic variation [20, 21] (Additional file 1: Fig. S1). *P* values were adjusted with the Benjamini–Hochberg correction to account for multiple testing, wherein the false discovery rate (FDR) was controlled at a 5% level.

### Genomic inflation estimation and correction

Quantile–Quantile (Q–Q) plots and genomic inflation factors (*λ*) were used to assess the inflation of *P* values for EWAS. Genomic correction was implemented with the Bacon method [22] to obtain inflation-corrected effect sizes, standard errors, and *P* values for each association, whereby significant CpGs were further filtered at a Bacon-corrected FDR < 0.05 level. Both the Bacon-corrected and conventional *λ*s were reported. Briefly, Bacon correction is a Bayesian-based method specifically designed to estimate and correct for bias and inflation of test statistics in EWAS, since it has been reported that the conventional λ often overestimates the actual test–statistic inflation [22]. The Bacon method was implemented using the *bacon* R package [22].

### Gene-set enrichment analysis

Nearest genes of the identified CpG sites were functionally annotated by the Functional Mapping and Annotation of GWAS (FUMA) GENE2FUNC online tool to investigate biological pathway enrichment [23]. All genes in Ensemblv92 were used for gene background with Genotype-Tissue Expression v8 representing 54 tissue types and 30 general tissue types. A minimum of two overlapping genes within gene sets was used with FDR-corrected *P* values < 0.05 considered as statistically significant.

### Sensitivity analysis

To examine whether use of hypoglycemic medications affects our results, we conducted sensitivity analysis by additional adjusting for use of hypoglycemic drugs (yes/no) in the above-described statistical models.

## Results

### Demographic and clinical characteristics of the participants

Demographic and clinical characteristics of the 75 individuals with severe obesity at baseline and 1-year follow-up are summarized in Table 1. The mean age at baseline was 51 ± 8 years, 12 (16%) participants were male, 34 (45%) had T2D and the mean BMI of the three groups ranged from 43.3 to 48.5 kg/m^2^. Rates of T2D, mean BMI, mean waist circumference (WC), FPG and HbA1C differed at baseline among the three intervention groups. At 1-year follow-up, T2D remission was observed in 4/7 (57%), 6/6 (100%) and 19/21 (90%) participants with baseline T2D from the IMI, BAND and RYGB groups, respectively. The three obesity parameters including BMI, body weight and WC, as well as cardio-metabolic biomarkers including blood pressure, FPG and HbA1c were significantly reduced in all intervention groups when compared with baseline (all *P* < 0.05).

**Table 1:** Characteristics of study participants before and 1-year after weight-loss intervention

Characteristics	IMI (*n* = 25)			BAND (*n* = 25)			RYGB (*n* = 25)			*p* for baseline difference
	Baseline	1-year	Change, Δ	Baseline	1-year	Change, Δ	Baseline	1-year	Change, Δ	
Age, years	51 ± 8	–	–	50 ± 8	–	–	51 ± 9	–	–	0.973
Sex, male (%)	4 (16)	–	–	4 (16)	–	–	4 (16)	–	–	1.000
Diabetes, yes (%)	7 (28)	3 (12)	4 (57)	6 (24)	0	6 (100)	21 (84)	2 (8)	19 (90)	< 0.001*
BMI, kg/m^2^	43.3 ± 6.5	38.3 ± 5.7	− 5.0 ± 3.3	45.7 ± 5.3	36.9 ± 5	− 8.8 ± 3.9	48.5 ± 6.2	31.2 ± 5.0	− 17.2 ± 4.8	0.013*
Weight, kg	120 ± 26	106 ± 21	− 14 ± 11	123 ± 16	100 ± 18	− 24 ± 10	133 ± 20	86 ± 16	− 47 ± 14	0.080
WC, cm	123 ± 15	115 ± 13	− 7 ± 8	130 ± 10	110 ± 12	− 20 ± 9	140 ± 11	101 ± 13	− 38 ± 12	< 0.001*
SBP, mmHg	126 ± 12	120 ± 17	− 6 ± 12	129 ± 15	115 ± 13	− 14 ± 19	129 ± 14	120 ± 19	− 10 ± 20	0.572
DBP, mmHg	82 ± 9	80 ± 8	− 2 ± 8	81 ± 8	73 ± 7	− 8 ± 8	78 ± 11	73 ± 9	− 5 ± 12	0.264
FPG, mg/dL	98 [90, 109]	93 [90, 98]	− 3 [− 7, 2]	103 [94, 110]	92 [88, 96]	− 11 [− 15, − 4]	118 [98, 155]	92 [84, 96]	− 27 [− 58, − 6]	0.044*
HbA1c, %	5.7 [5.5, 6.1]	5.3 [5.2, 5.6]	− 0.4 [− 0.5, − 0.2]	5.9 [5.8, 6.1]	5.4 [5.2, 5.6]	− 0.5 [− 0.6, − 0.3]	7.2 [6.4, 9.1]	5.5 [5.3, 5.8]	− 1.3 [− 3.1, − 0.8]	< 0.001*

Post-intervention change was tested by paired *t* test or Wilcoxon rank test. All *P* values were less than 0.05. Difference in baseline characteristics among groups were tested by one-way ANOVA, Chi-square test or Fisher’s exact test. *Indicates statistical significance (*P* ≤ 0.05). IMI—intensive medical intervention; BAND—gastric banding; RYGB—Roux-en-Y gastric bypass; BMI—body mass index; WC—waist circumference; SBP—systolic blood pressure; DBP—diastolic blood pressure; FPG—fasting plasma glucose; HbA1c—hemoglobin A1c. Categorical variables were expressed as frequency (percentage) and continuous variables as mean ± SD, except FPG and HbA1c as median [Q25, Q75]

### Differential associations between DNAm and glycemic outcomes in response to different types of weight-loss intervention

**Fig. 2:**
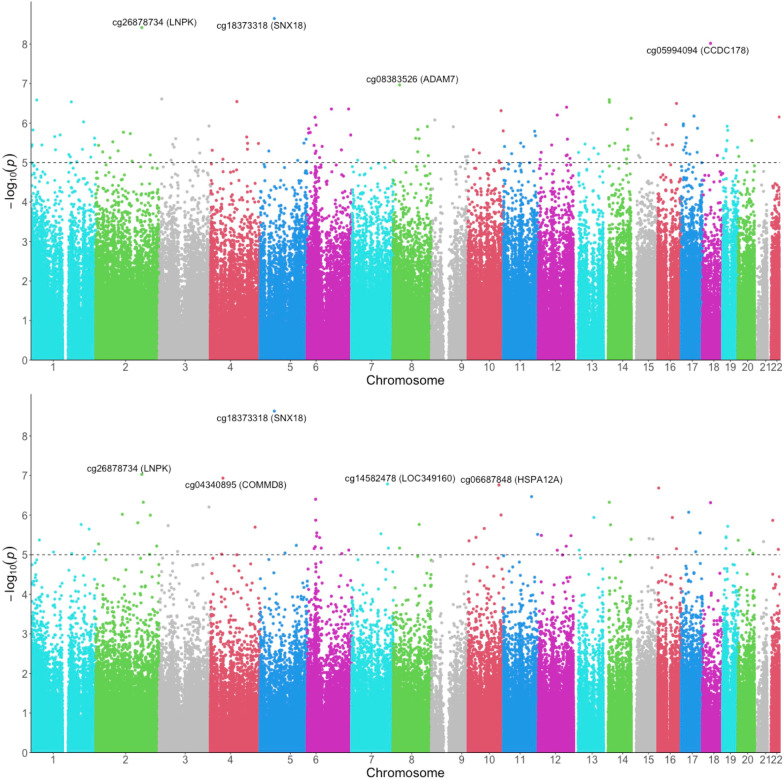
Manhattan plots depicting CpGs differentially associated with changes in glycemic measures, RYGB versus IMI. Baseline DNA methylation was differentially associated with changes in FPG (top), and HbA1c (bottom). The dashed line represents the suggestive significance threshold of *P* = 1.0 × 10^−5^. Abbreviations: FPG—fasting plasma glucose; HbA1c—hemoglobin A1c; IMI—intensive medical intervention; RYGB—Roux-en-Y gastric bypass

After adjustment for age, sex, post-intervention weight loss, baseline BMI, baseline FPG or HbA1c and the surrogate variable, baseline DNAm levels at 3216 and 117 CpG loci were differentially associated with changes in FPG and HbA1c, respectively, when comparing RYGB versus IMI groups (FDR for interaction < 0.05). The differential associations of DNAm were not seen in other group comparisons (BAND vs. IMI or RYGB vs. BAND), except for 6 CpGs identified for HbA1c changes when comparing RYGB versus BAND. Figure 2 shows the Manhattan plots of CpGs differentially associated with glycemic outcomes one year after RYGB versus IMI (Manhattan plots for the BAND versus IMI and RYGB vs. BAND comparisons can be found in Additional file 1: Fig. S2). For the RYGB versus IMI comparison, 79 CpG sites were significant for changes in both FPG and HbA1c, among which the top 20 significant sites ranked by *P* values from the FPG models are shown in Table 2.

**Table 2:** Top 20 (of 79) CpGs differentially associated with changes in glycemic measures, RYGB versus IMI

CpG	CHR	Position	Nearest gene	Relation to CpG islands	Change in FPG					Change in HbA1c				
					*β* (RYGB)	SE (RYGB)	*β* (IMI)	SE(IMI)	FDR for interaction	*β* (RYGB)	SE (RYGB)	*β* (IMI)	SE (IMI)	FDR for interaction
cg18373318	5	53813164	SNX18	N_Shore	− 83.3	70.0	733.8	111.6	0.001	− 2.2	2.1	23.8	3.5	0.001
cg26878734	2	176793622	LNPK	Sea	− 481.5	200.6	1244.9	218.0	0.001	− 12.7	6.0	36.8	7.0	0.009
cg05994094	18	31020806	CCDC178	Island	− 282.0	150.3	1474.4	253.4	0.001	− 3.2	4.7	46.5	8.2	0.012
cg08383526	8	24297818	ADAM7	Sea	656.5	229.9	− 1253.1	248.6	0.007	13.8	7.4	− 37.8	8.1	0.033
cg21191176	14	23402052	PRMT5	S_Shelf	766.9	461.3	− 3525.3	531.4	0.008	34.1	14.8	− 97.2	17.2	0.012
cg18941458	1	22191585	HSPG2	Island	884.1	463.1	− 2299.7	496.8	0.008	15.0	14.7	− 73.1	16.3	0.041
cg03831971	4	100868132	DNAJB14	Island	− 1852.7	511.8	3392.9	776.4	0.008	− 40.1	16.5	104.2	25.3	0.037
cg00658652	16	71500215	ZNF23	S_Shelf	225.7	114.8	− 620.4	119.2	0.008	7.5	3.8	− 16.5	4.0	0.033
cg22964496	10	126718276	CTBP2	S_Shelf	735.9	291.5	− 1383.4	314.7	0.009	17.8	8.9	− 46.7	9.8	0.017
cg08691332	12	72142653	–	Sea	745.5	341.7	− 1578.2	361.5	0.01	22.4	10.9	− 43.8	11.8	0.033
cg18144742	14	107252,125	–	Sea	126.7	245.8	1880.7	347.8	0.01	10.1	7.5	62.9	10.9	0.026
cg06876872	1	202110290	ARL8A	N_Shelf	− 2234.6	496.9	1146.7	428.5	0.011	− 35.9	14.6	57.0	13.6	0.04
cg03863499	3	191670165	–	Sea	210.3	350.8	− 2691.8	435.8	0.011	19.7	11.5	− 74.4	14.0	0.014
cg14580737	19	19301780	BORCS8-MEF2B	N_Shore	302.0	153.8	− 660.0	158.8	0.012	16.4	4.8	− 13.5	5.0	0.02
cg05310882	15	83378778	AP3B2	Island	− 239.9	853.5	8075.1	1537.8	0.012	13.7	26.1	267.0	47.1	0.026
cg11971662	8	99499993	STK3	Sea	875.3	411.0	− 1733.9	341.0	0.012	25.9	12.5	− 55.7	10.4	0.02
cg01750895	17	17463041	PEMT	N_Shelf	1181.4	304.7	− 555.4	210.3	0.013	28.6	9.0	− 20.1	6.6	0.047
cg13288301	3	52446425	BAP1	S_Shore	− 683.2	299.4	1678.8	433.0	0.013	− 14.4	8.9	55.8	14.0	0.045
ch.16.54217905R	16	55660404	–	Sea	9355.0	2009.4	− 1675.1	1066.5	0.013	261.9	56.0	− 74.2	32.2	0.017
cg13057898	1	3703894	LRRC47	Island	300.4	100.9	− 278.1	81.4	0.013	6.5	3.1	− 10.2	2.6	0.045

*CHR*—chromosome, *FDR*—false discovery rate, *FPG*—fasting plasma glucose, *HbA1c*—hemoglobin A1c, *SE*—standard error

**Fig. 3:**
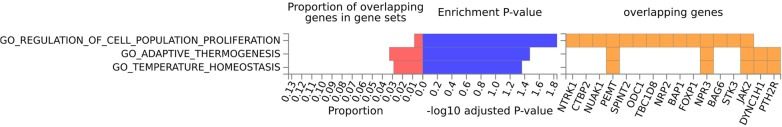
Functional enrichment of genes differentially associated with changes in glycemic measures, RYGB versus IMI. Gene sets were derived from the biological process category of gene ontology in MSigDB. Abbreviations: IMI, intensive medical intervention; RYGB, Roux-en-Y gastric bypass

Enrichment analysis was conducted for the 79 CpG sites identified from the RYGB versus IMI comparison. They were annotated to 68 nearby genes, which are involved in three biological processes: adaptive thermogenesis, temperature homeostasis and regulation of cell population proliferation, from the biological process category of gene ontology in MSigDB. No enriched pathways were found for hallmark gene sets. More detailed information of gene pathways identified is shown in Fig. 3.

The top 20 CpG sites differentially associated with changes in glycemic measures for RYGB versus IMI, BAND versus IMI, and RYGB versus BAND comparisons can be found in Additional file 2: Tables S1 to S3.

### Genomic correction

Among the 3216 CpGs that were differentially associated with FPG change when comparing RYGB versus IMI, four CpGs remained significant after Bacon correction, i.e., cg18373318 [Sorting nexin 18 (*SNX18)*], cg26878734 [Lunapark, ER junction formation factor (*LNPK*)], cg05994094 [Coiled-coil domain-containing protein 178 (*CCDC178)*] and cg08383526 [ADAM metallopeptidase domain 7 (*ADAM7)*]. Meanwhile, out of 117 CpGs that were differentially associated with HbA1c change when comparing RYGB versus IMI, 84 CpGs survived Bacon correction; this set included the 4 significant CpGs associated with FPG change after Bacon correction. However, neither Bacon-surviving set of CpGs showed significant enrichment for any gene ontology pathway. Additionally, although no significant CpGs were identified for FPG when comparing RYGB versus BAND, the 6 CpGs that were associated with change in HbA1c all survived Bacon correction. More detailed information of Bacon-corrected significant CpGs is shown in Additional file 2: Table S4.

The Bacon-corrected *λ* values ranged from 0.9975 to 1.2073. Q–Q plots and λs generated by both Bacon correction and the conventional approach are shown in Additional file 1: Fig. S3.

### Results for sensitivity analysis

After further adjustment for use of hypoglycemic medications, 669 CpGs (out of 3216 CpGs) and 71 CpGs (out of 117 CpGs) remained statistically significant at FDR < 0.05 for changes in FPG and HbA1c, respectively, when comparing RYGB versus IMI. Results for these analyses are shown in Supplemental Table S5. The total number of significant CpGs remained unchanged for both glycemic outcomes when comparing RYGB versus BAND or BAND versus IMI.

## Discussion

To our knowledge, this is the first study to date demonstrating that baseline blood DNAm is differentially associated with changes in glycemic measures induced by different types of weight-loss interventions. When comparing patients undergoing RYGB surgery with those that followed the non-surgical IMI, a number of CpG sites were identified to be differentially associated with change in FPG and HbA1c, mapping to genes that are implicated in adaptive thermogenesis, temperature homeostasis and regulation of cell population proliferation. These findings suggest that baseline DNAm may explain, at least partially, the large variability in glycemic outcomes induced by different weight-loss interventions.

Among the identified significant CpG sites differentially associated with changes in both FPG and HbA1c (RYGB vs. IMI), 4 CpG sites including cg18373318 (*SNX18*), cg26878734 (*LNPK*), cg05994094 (*CCDC178*) and cg08383526 (*ADAM7*) showed robust significance after genomic correction. Interestingly, methylation at cg18373318 (*SNX18*) has been previously shown to be associated with ischemic stroke by a previous EWAS [24]; ischemic stroke is one of the multiple cardiovascular outcomes observed in individuals with obesity [25]. The *SNX18* gene encodes a member of the sorting nexin family, which are involved in endocytosis and intracellular vesicle trafficking [26]. Dysfunctions affecting the sorting nexin pathway have been reported to be involved in cardiovascular disease and related risk factors, including hypertension, heart failure and coronary artery aneurysm [27]. In addition, methylation at cg08383526 (*ADAM7*) was previously associated with T2D at a nominal significance level of *P* < 0.05 (*P* = 2.32 × 10^–4^) [28]. *ADAM7* encodes a member of the ADAMs family of zinc proteases. Even though there is still limited knowledge about the role of ADAMs in cardiovascular pathologies, previous research showed that several members of the ADAMs family were involved in cardiovascular development or cardiomyopathies [29]. Meanwhile, cardiometabolic outcomes have not been previously associated with methylation at either cg26878734 (*LNPK*) or cg05994094 (*CCDC178*). Notably, cg26878734 (*LNPK*) was previously associated with rheumatoid arthritis in a monozygotic twin study [30], suggesting its involvement in inflammation, while genetic variability at the *CCDC178* gene has been associated with body fat distribution in a genome-wide association study [31].

The mechanisms linking DNAm to differential glycemic response to obesity interventions are yet to be elucidated; however, our findings revealed some biologically plausible pathways. Adaptive thermogenesis, defined as a greater than expected reduction in resting metabolic rate (RMR), has been previously reported as a contributor to unsuccessful weight control [32]. An excessive RMR reduction has been proposed as a defense mechanism aimed at protecting energy stores in the face of starvation; thus, individuals with obesity experiencing a dramatic weight loss after surgery are predisposed to weight regain via a lowered RMR. Pathway enrichment analysis of the identified CpGs in the present study showed an over-representation of adaptive thermogenesis, implying that defense against this process may contribute to the long-lasting weight loss and metabolic improvement induced by RYGB. In line with our findings, one study assessed the changes in RMR in adults with severe obesity six months after RYGB (*n* = 8) and BAND (*n* = 5), and observed minimal adaptive thermogenesis following RYGB [33]. The lack of evidence for adaptive thermogenesis following RYGB or BAND was also supported by other studies [34, 35]. In addition, many studies have associated temperature homeostasis with bariatric surgery outcomes through the thermoregulatory role of brown adipose tissue (BAT) [36, 37]. For instance, RYGB caused selective activation of BAT to induce thermogenesis upon cold exposure in mice, independent of weight loss [37]. Such increased activity of BAT may contribute to increased energy expenditure and weight loss maintenance after bariatric surgery. Although BAT thermogenesis is not the focus of the present study, our enrichment analysis of differentially methylated probes is in agreement with the involvement of temperature homeostasis in bariatric surgery-induced weight loss. Lastly, the identified significant CpG sites in our study were also enriched in cell population proliferation, which in the context of obesity may involve various cell types, such as pancreatic β-cells, intestinal epithelial cells and hepatocytes, among others. Pancreatic β-cells secrete insulin in response to glucose levels. Non-diabetic subjects with obesity have been reliably described to exhibit increased β-cell mass when compared to lean individuals [38]; however, diabetes is characterized by a decrease in β-cell mass [39]. These contrasting findings suggest that different rates of pancreatic β-cell proliferation are present in subjects with obesity as a function of their diabetic status [40]. Rectal epithelial cell proliferation has been described to be persistently elevated at three years post-RYGB among human subjects [41], while intestinal crypt cell proliferation has been proposed as a possible mechanism to prevent malabsorption despite the reduced intestinal surface available after RYGB [42]. Obesity is a risk factor for non-alcoholic fatty liver disease, which leads to hepatocyte proliferation in response to liver damage; increased hepatocyte proliferation has been reported both in a mice model of obesity [43] and associated with mice ghrelin levels [44]. Together, these findings point to the involvement of cell proliferation of different cell populations in both obesity and response to bariatric surgery. Pre-existing DNAm at CpG sites involved in adaptive thermogenesis, temperature homeostasis, and regulation of cell proliferation may underlie the differential effects of weight-loss interventions on glycemic outcomes.

The present study has several strengths. The innovative study design allowed us to identify baseline DNAm signatures that are differentially associated with glycemic outcomes induced by different types of weight-loss intervention. The observed associations with glycemic outcomes are independent of weight loss and baseline clinical factors. Moreover, both FPG and HbA1c were included in the analyses as the glycemic outcomes of interest and the overlap between both sets of statistical models was used to increase the robustness of the results. However, some limitations should also be noted. First, our sample size is small, and thus we might only detect CpGs with large effect size. Those with small and modest effect size may have been missed out. In addition, due to lack of an external cohort with a similar study design and comparable datasets used in the current analysis, we were unable to replicate our findings. Further investigation is warranted to confirm our results in larger populations. Second, due to ethical and clinical reasons, participants could not be randomized but instead they were enrolled in different treatment groups based on clinical characteristics and personal preferences. This may lead to baseline differences among the groups. However, this should not be a concern for our study because we have adjusted for these clinical factors in all statistical models. Third, DNAm was measured in blood samples but not the target organ of obesity (e.g., adipose tissue). Nevertheless, blood sample is of clinical relevance to the identification of non-invasive biomarkers in many previous studies. Forth, there was a potential genomic inflation based on the conventional *λ*s. However, *P* value inflation is commonly observed in many DNAm studies [22, 45, 46] and standard approaches to control for inflation in genome-wide association studies are not suitable for EWAS data [22, 46]. Nonetheless, we used a stringent significance threshold to control for false positive rate, as well as genomic correction with the Bacon method to mitigate the effects of potential inflation and bias. Fifth, because HeadsUp did not collect PAXgene tubes for gene expression analysis, we were unable to functionally validate the differentially methylated genes identified in our EWAS. Nevertheless, we performed functional enrichment analysis to explore the possible biological pathways for the putative candidate genes.

## Conclusion

In summary, our results suggest that (1) DNAm alteration may underlie the mechanism through which different types of weight-loss intervention may exert differential glycemic outcomes, independent of weight loss; and (2) baseline DNAm may serve as potential biomarkers for identifying individuals who would benefit the most from specific types of bariatric surgery. However, given the small sample size and the observational nature of our study, more research is needed to confirm our findings.

## Supplementary Information

**Additional file 1: Fig. S1**. Heatmap showing the correlations between surrogate variableand cell type compositions. **Fig. S2**. Manhattan plots depicting CpGs differentially associated with glycemic outcomes in response to BAND vs. IMI and RYGB vs. BAND. **Fig. S3**. Q–Q plots of p-values and λ for the epigenome-wide differential associations between DNA methylation and glycemic outcomes, Bacon-correctedvs. conventional approach.

**Additional file 2: Table S1**. Top 20 CpGs differentially associated with changes in glycemic measures, RYGB vs. IMI. **Table S2**. Top 20 CpGs differentially associated with changes in glycemic measures, BAND vs. IMI. **Table S3**. Top 20 CpGs differentially associated with changes in glycemic measures, RYGB vs. BAND. **Table S4**. CpGs differentially associated with changes in glycemic measures, after Bacon correction. **Table S5**. Top 20 CpGs differentially associated with changes in glycemic measures, RYGB vs. IMI.

## Data Availability

Data used in this study can be obtained from the corresponding author upon a reasonable request.
