# Supplementary material for: DNA methylation is differentially associated with glycemic outcomes by different types of weight-loss interventions: an epigenome-wide association study
Source: Clin Epigenetics. 2023 Jul 1;15:108. doi: 10.1186/s13148-023-01522-9 (PMC10314401; doi:10.1186/s13148-023-01522-9)
Supplement: Supplementary file 1 — Additional file 1: Fig. S1. Heatmap showing the correlations between surrogate variableand cell type compositions. Fig. S2. Manhattan plots depicting CpGs differentially associated with glycemic outcomes in response to BAND vs. IMI and RYGB vs. BAND. Fig. S3. Q–Q plots of p-values and λ for the epigenome-wide differential associations between DNA methylation and glycemic outcomes, Bacon-correctedvs. conventional approach. [file 13148_2023_1522_MOESM1_ESM.docx]

| **Supplemental Figure S1**. Heatmap showing the correlations between surrogate variable (V1) and cell type compositions | |
| --- | --- |
| 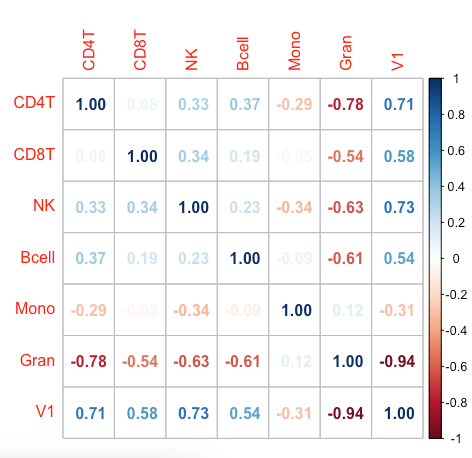 |  |

**Supplemental Figure S2**. Manhattan plots depicting CpGs differentially associated with glycemic outcomes in response to BAND vs. IMI and RYGB vs. BAND.

| 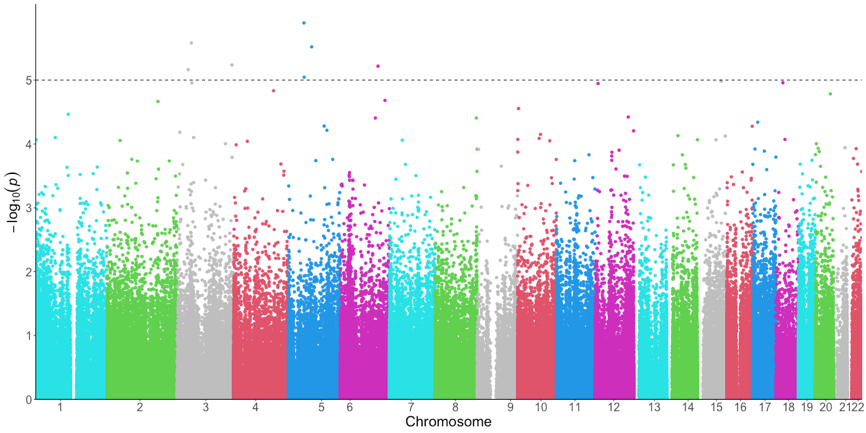  A. CpGs associated with change in FPG, BAND vs. IMI | 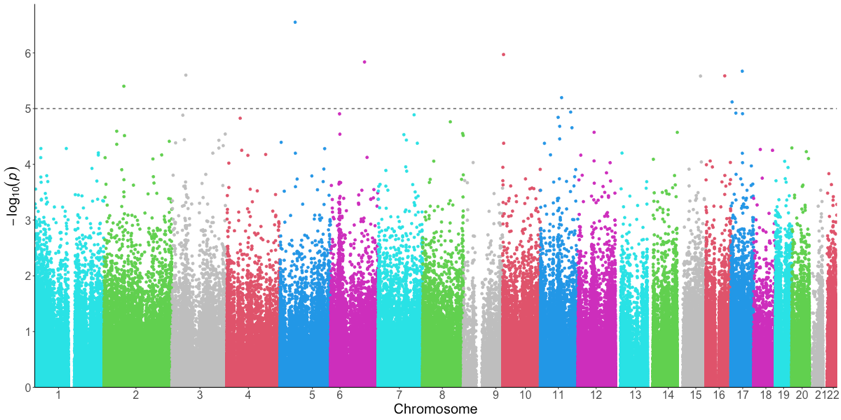  C. CpGs associated with change in HbA1c, BAND vs. IMI |
| --- | --- |
| 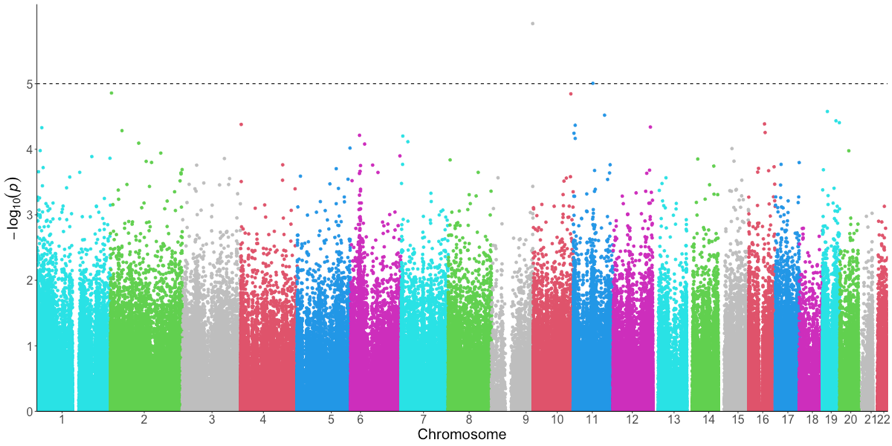  B. CpGs associated with change in FPG, RYGB vs. BAND | 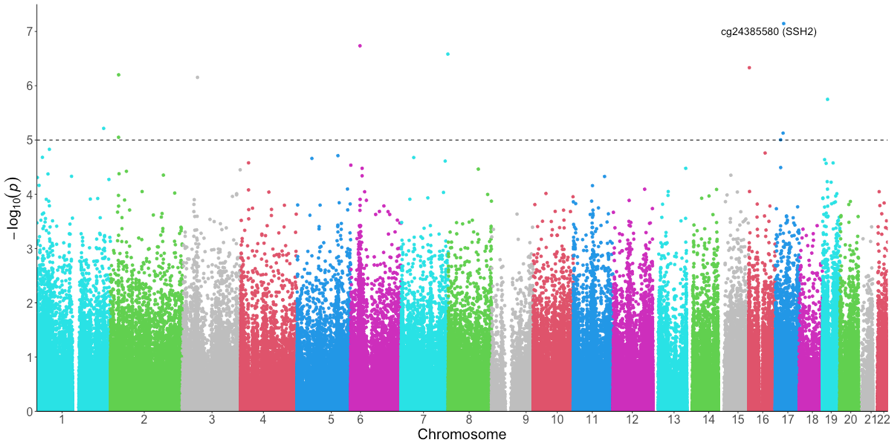  D. CpGs associated with change in HbA1c, RYGB vs. BAND |
| The dashed line represents the suggestive significance threshold of P = 1.0×10^−5^; Abbreviations: BAND, gastric banding; IMI, intensive medical intervention; RYGB, Roux-en-Y gastric bypass; FPG, fasting plasma glucose; HbA1c, hemoglobin A1c. | |

**Supplemental Figure S3**. Q-Q plots of p-values and λ for the epigenome-wide differential associations between DNA methylation and glycemic outcomes, Bacon-corrected(top) vs. conventional approach(bottom).

| 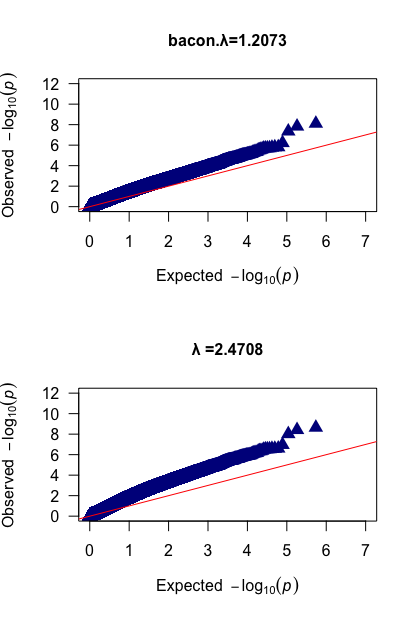  A. FPG change, RYGB vs. IMI | 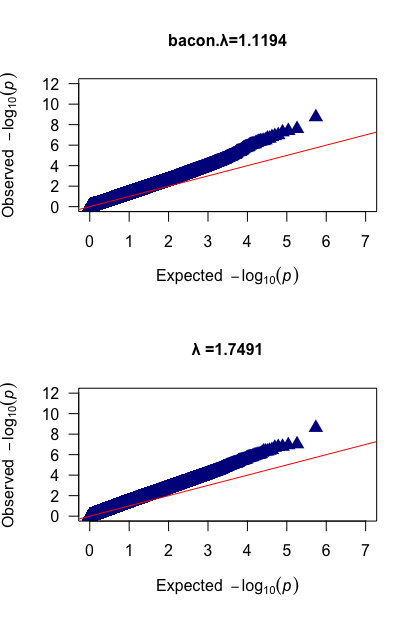  D. HbA1c change, RYGB vs. IMI | |
| --- | --- | --- |
| 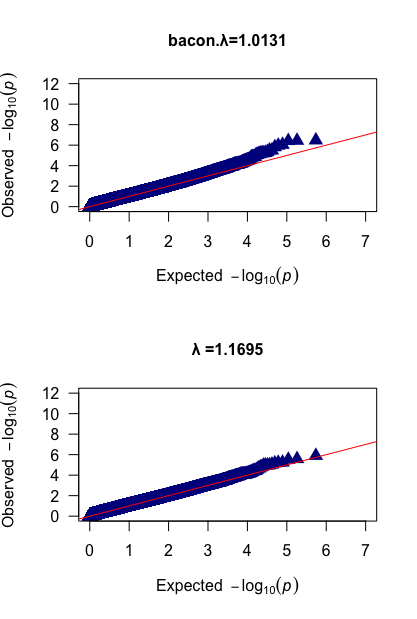  B. FPG change, BAND vs. IMI | 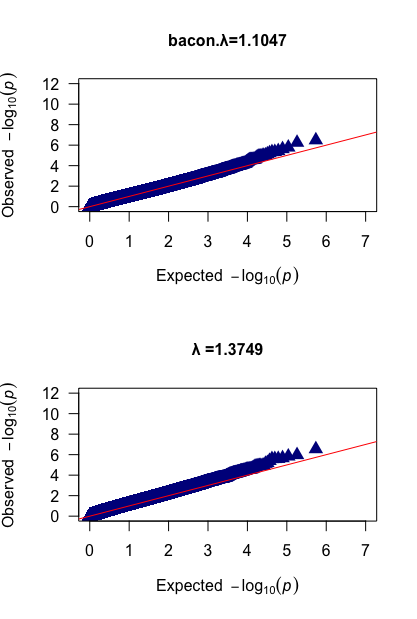  E. HbA1c change, BAND vs. IMI | |
| 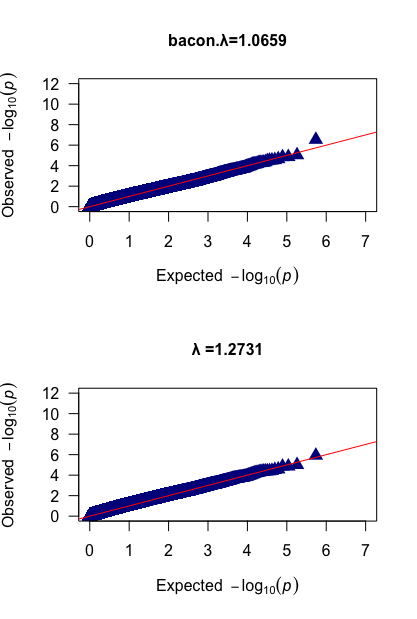  C. FPG change, RYGB vs. BAND | | 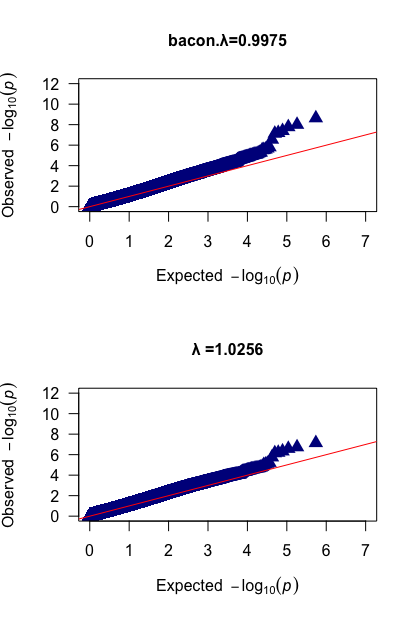  F. HbA1c change, RYGB vs. BAND |

Abbreviations: BAND, gastric banding; IMI, intensive medical intervention; RYGB, Roux-en-Y gastric bypass; FPG, fasting plasma glucose; HbA1c, hemoglobin A1c.
