# Supplementary material for: DNA methylation is differentially associated with glycemic outcomes by different types of weight-loss interventions: an epigenome-wide association study
Source: Clin Epigenetics. 2023 Jul 1;15:108. doi: 10.1186/s13148-023-01522-9 (PMC10314401; doi:10.1186/s13148-023-01522-9)
Supplement: Supplementary file 2 — Additional file 2: Table S1. Top 20 CpGs differentially associated with changes in glycemic measures, RYGB vs. IMI. Table S2. Top 20 CpGs differentially associated with changes in glycemic measures, BAND vs. IMI. Table S3. Top 20 CpGs differentially associated with changes in glycemic measures, RYGB vs. BAND. Table S4. CpGs differentially associated with changes in glycemic measures, after Bacon correction. Table S5. Top 20 CpGs differentially associated with changes in glycemic measures, RYGB vs. IMI. [file 13148_2023_1522_MOESM2_ESM.pdf]

Supplemental Tables:

Supplemental Table S1. Top 20 CpGs differentially associated with changes in glycemic measures, RYGB vs. IMI
Supplemental Table S2. Top 20 CpGs differentially associated with changes in glycemic measures, BAND vs. IMI
Supplemental Table S3. Top 20 CpGs differentially associated with changes in glycemic measures, RYGB vs. BAND
Supplemental Table S4. CpGs differentially associated with changes in glycemic measures, after Bacon correction
Supplemental Table S5. Top 20 CpGs differentially associated with changes in glycemic measures, RYGB vs. IMI (sensitivity analyses)

Supplemental Table S1. Top 20 CpGs differentially associated with changes in glycemic measures, RYGB vs. IMI

CpG	CHR	Position	Nearest gene	Relation to CpG islands	β (RYGB)	SE(RYGB)	β (IMI)	SE(IMI)	*p* for interaction	FDR for interaction
Change in FPG										
cg18373318	5	53813164	SNX18	N_Shore	-83.3	70	733.8	111.6	2.26E-09	0.001
cg26878734	2	176793622	LNPK	Sea	-481.5	200.6	1244.9	218	3.82E-09	0.001
cg05994094	18	31020806	CCDC178	Island	-282	150.3	1474.4	253.4	9.61E-09	0.001
cg08383526	8	24297818	ADAM7	Sea	656.5	229.9	-1253.1	248.6	1.09E-07	0.007
cg01139906	3	10290027	IRAK2	Island	-856.4	340.5	2423.7	481	2.45E-07	0.008
cg21191176	14	23402052	PRMT5	S_Shelf	766.9	461.3	-3525.3	531.4	2.58E-07	0.008
cg18941458	1	22191585	HSPG2	Island	884.1	463.1	-2299.7	496.8	2.61E-07	0.008
cg03831971	4	100868132	DNAJB14	Island	-1852.7	511.8	3392.9	776.4	2.86E-07	0.008
cg26232102	1	155024228	DCST1	Island	-2723.1	798	3035.2	679	2.91E-07	0.008
cg19512521	14	23821902	SLC22A17	Island	-4176.2	2006.7	10324.1	1833.2	2.96E-07	0.008
cg00658652	16	71500215	ZNF23	S_Shelf	225.7	114.8	-620.4	119.2	3.19E-07	0.008
cg27431274	12	107979028	BTBD11	S_Shelf	642.2	155.7	-407.9	158	3.97E-07	0.009
cg03467087	6	158448134	SYNJ2	Sea	-330.7	119.1	576	129.5	4.40E-07	0.009
cg23724489	6	92401486	-	Sea	854.6	194.2	-412.6	160.3	4.41E-07	0.009
cg22964496	10	126718276	CTBP2	S_Shelf	735.9	291.5	-1383.4	314.7	4.87E-07	0.009
cg08691332	12	72142653	-	Sea	745.5	341.7	-1578.2	361.5	6.29E-07	0.01
cg22002216	17	48502923	ACSF2	N_Shore	-431.9	218	1256.4	259.1	6.65E-07	0.01
cg08503964	22	47370307	TBC1D22A	Island	1097.8	418.9	-2120.1	454.9	7.01E-07	0.01
cg20496034	6	29574810	GABBR1	Sea	2351	731.7	-1902.2	440.5	7.14E-07	0.01
cg18144742	14	107252125	-	Sea	126.7	245.8	1880.7	347.8	7.56E-07	0.01
Change in HbA1c										
cg18373318	5	53813164	SNX18	N_Shore	-2.2	2.1	23.8	3.5	2.36E-09	0.001
cg26878734	2	176793622	LNPK	Sea	-12.7	6	36.8	7	9.37E-08	0.009
cg04340895	4	47463168	COMMD8	N_Shelf	24.8	11.7	-87.8	15	1.16E-07	0.009
cg14582478	7	136845265	LOC349160	N_Shelf	83.4	20.3	-59.4	14.4	1.64E-07	0.009
cg06687848	10	118547556	HSPA12A	N_Shore	-12.7	7.1	42.3	7.9	1.73E-07	0.009
cg08336593	16	3280662	ZNF200	N_Shelf	95.4	22.5	-62.9	16.4	2.07E-07	0.009
cg05081395	11	108345522	KDELC2	Sea	42.1	22.1	-148.2	27.4	3.41E-07	0.012
cg17619093	6	31620354	BAG6	Island	521.7	86.6	-21.9	41.9	3.98E-07	0.012
cg21995919	2	182322279	ITGA4	Island	-467.7	91.5	225.5	92.1	4.73E-07	0.012
cg21191176	14	23402052	PRMT5	S_Shelf	34.1	14.8	-97.2	17.2	4.74E-07	0.012
cg05994094	18	31020806	CCDC178	Island	-3.2	4.7	46.5	8.2	4.84E-07	0.012
cg03863499	3	191670165	-	Sea	19.7	11.5	-74.4	14	6.25E-07	0.014
cg24385580	17	28088301	SSH2	Island	158.2	26.9	-4.3	11.2	8.48E-07	0.017
cg01850334	2	101667004	TBC1D8	Sea	33.7	11.7	-58.7	14.1	9.55E-07	0.017
cg22964496	10	126718276	CTBP2	S_Shelf	17.8	8.9	-46.7	9.8	9.93E-07	0.017
cg23154849	2	209240193	PTH2R	Sea	14.4	5.1	-19.4	6	1.00E-06	0.017
cg09010671	13	79177763	RNF219-AS1	Island	-57.3	29.4	194.9	40.2	1.14E-06	0.017
ch.16.54217905R	16	55660404	-	Sea	261.9	56	-74.2	32.2	1.15E-06	0.017
cg25549720	6	32058851	TNXB	S_Shelf	32.8	8.6	-18.4	6.9	1.35E-06	0.019
cg04282607	22	22007286	MIR301B	S_Shore	23.6	4.7	-7	3.8	1.36E-06	0.019

Abbreviations: RYGB, Roux-en-Y gastric bypass; IMI, intensive medical intervention; CHR, chromosome; SE, standard error; FDR, false discovery rate; FPG, fasting plasma glucose; HbA1c, hemoglobin A1c.

Supplemental Table S2. Top 20 CpGs differentially associated with changes in glycemic measures, BAND vs. IMI

CpG	CHR	Position	Nearest gene	Relation to CpG islands	β (BAND)	SE(BAND)	β (IMI)	SE(IMI)	*p* for interaction	FDR for interaction
Change in FPG										
cg18373318	5	53813164	SNX18	N_Shore	55	67.5	733.8	111.6	1.27E-06	0.273
cg12691534	3	50275394	GNAI2	Island	-418.2	537.5	4683.1	855.3	2.61E-06	0.273
cg01491225	5	80597551	RNU5E-1	Island	-902.4	1200.6	12182.5	2292.4	3.01E-06	0.273
cg03863499	3	191670165	-	Sea	-102.7	326	-2691.8	435.8	5.78E-06	0.28
cg04600795	6	130774523	-	Sea	-3.9	14.9	-736.9	147.2	6.04E-06	0.28
cg13621925	3	39423611	SLC25A38	N_Shore	-100	167	1725.1	345.4	6.84E-06	0.28
cg00089719	5	54527320	MCIDAS	Island	-249.3	474.6	3619.5	682.8	8.98E-06	0.28
cg05310882	15	83378778	AP3B2	Island	150.6	952.9	8075.1	1537.8	1.04E-05	0.28
cg03740978	18	24127875	KCTD1	Island	-12.6	1038.9	8398.4	1445.9	1.10E-05	0.28
cg22621867	3	51990301	GPR62	Island	5.9	263.3	2438.8	453	1.11E-05	0.28
cg15909016	12	9800818	LOC374443	Island	-588	1385.5	10001.9	1863.4	1.13E-05	0.28
cg06712846	4	138864252	-	Sea	-142.3	131.1	772.7	146.8	1.46E-05	0.332
cg12032027	20	52198225	ZNF217	Island	-245.3	84.9	-765.9	112.1	1.64E-05	0.344
cg24794206	6	155316492	TIAM2	Island	-78.8	491.1	4312.6	854.4	2.08E-05	0.393
cg26878734	2	176793622	LNPK	Sea	73.8	121	1244.9	218	2.16E-05	0.393
cg02773337	10	3171970	PFKP	Sea	-217.8	1207.2	-9198.1	1562.5	2.79E-05	0.475
cg00963079	1	113499149	SLC16A1	Island	-65.5	451.6	3513	721.5	3.42E-05	0.534
cg26385172	12	115135169	-	N_Shore	-68.2	155.3	1021.8	205.7	3.78E-05	0.534
cg26332253	6	121758729	GJA1	Sea	14.4	158.8	1228.8	246.5	3.91E-05	0.534
cg08860119	8	143695901	ARC	Island	-47	233.8	2510.4	539.3	3.92E-05	0.534
Change in HbA1c										
cg18373318	5	53813164	SNX18	N_Shore	1.3	2.1	23.8	3.5	2.82E-07	0.077
cg02773337	10	3171970	PFKP	Sea	11.7	35.7	-303.3	45.8	1.07E-06	0.102
cg26332253	6	121758729	GJA1	Sea	-1.6	4.7	41.1	7.3	1.46E-06	0.102
cg06378498	17	40540460	STAT3	Island	-4.5	4.3	41.1	7.8	2.13E-06	0.102
cg12691534	3	50275394	GNAI2	Island	-10.2	16.5	146.7	26.1	2.51E-06	0.102
cg01114989	16	67757411	GFOD2	S_Shelf	7.3	8.7	-67.5	12.1	2.59E-06	0.102
cg05310882	15	83378778	AP3B2	Island	12.2	29.5	267	47.1	2.61E-06	0.102
cg14784608	2	70781101	TGFA	Island	-53.9	55	452.8	87.7	3.97E-06	0.135
cg21238457	11	75917353	WNT11	Island	-3.4	11.3	79.8	14.9	6.36E-06	0.193
cg15071166	17	3771325	CAMKK1	Island	0.3	0.8	-5.8	1	7.60E-06	0.207
cg05081395	11	108345522	KDELC2	Sea	16.7	21.7	-148.2	27.4	1.16E-05	0.224
cg08745599	17	17495332	PEMT	Island	-5.8	65.7	522.6	87.8	1.21E-05	0.224
cg21506159	17	41466287	LINC00910	Sea	-59.2	27.4	140.4	31.9	1.23E-05	0.224
cg12616421	6	32017224	TNXB	Sea	6.4	15.3	-110.8	21.2	1.24E-05	0.224
cg17998566	7	128478670	FLNC	N_Shelf	12.2	6.8	-49.4	11.2	1.29E-05	0.224
cg13621925	3	39423611	SLC25A38	N_Shore	-4.7	4.9	46.4	10.1	1.31E-05	0.224
cg14023073	11	63591940	SPINDOC	Sea	2.5	9.5	-83.9	15.8	1.44E-05	0.225
cg04340895	4	47463168	COMMD8	N_Shelf	-3.2	10.6	-87.8	15	1.49E-05	0.225
cg11971662	8	99499993	STK3	Sea	2.4	8.6	-55.7	10.4	1.73E-05	0.248
cg15244778	11	69048637	-	Sea	9.1	15.5	-110.2	21.4	2.08E-05	0.277

Abbreviations: BAND, gastric banding; IMI, intensive medical intervention; CHR, chromosome; SE, standard error; FDR, false discovery rate; FPG, fasting plasma glucose; HbA1c, hemoglobin A1c.

Supplemental Table S3. Top 20 CpGs differentially associated with changes in glycemic measures, RYGB vs. BAND

CpG	CHR	Position	Nearest gene	Relation to CpG islands	β (RYGB)	SE(RYGB)	β (BAND)	SE(BAND)	*p* for interaction	FDR for interaction
Change in FPG										
cg13764516	9	139648911	LOC100128593	Sea	-1112.3	173.8	-42.3	96.9	1.21E-06	0.329
cg01183579	11	66512052	C11orf80	Island	16635.8	3080.2	-1378.2	1880.4	9.84E-06	0.830
cg23275064	2	3749979	ALLC	N_Shore	801.1	159.4	-19	93.4	1.39E-05	0.830
cg02533235	10	127512031	UROS	Island	17362.3	2994.4	-206.8	2111.7	1.43E-05	0.830
cg08563299	19	16922592	NWD1	S_Shelf	703.7	145	-74.9	90.1	2.65E-05	0.830
cg04891917	11	105947743	KBTBD3	N_Shore	7167	1414.3	-585.3	940.6	3.01E-05	0.830
cg07765912	19	45954356	ERCC1	Island	10333.2	2148.7	-1435.7	1343.1	3.68E-05	0.830
cg15473155	19	57349204	ZIM2	N_Shore	-909.7	171.7	-22.7	126.7	3.93E-05	0.830
cg10965432	16	53537025	AKTIP	Island	9291.2	1893.4	-1161.3	1401.6	4.10E-05	0.830
cg25112220	4	1737051	TACC3	Island	4181.9	761.8	387	449	4.18E-05	0.830
cg26146306	11	6633474	ILK	Island	30952.7	6496.6	-2118.6	3729.8	4.30E-05	0.830
cg17389956	12	126167667	-	N_Shore	457.4	92.9	-36.6	63.4	4.57E-05	0.830
cg23107144	1	17196838	CROCC	N_Shore	-2136.3	438	325.1	343	4.69E-05	0.830
cg03184472	2	39174738	ARHGEF33	Sea	-5212.1	971.5	150.7	737.1	5.20E-05	0.830
ch.16.54217905R	16	55660404	-	Sea	9355	2009.4	-87.2	831.6	5.55E-05	0.830
cg24492694	11	2595605	KCNQ1	N_Shore	2122.8	393.9	233.1	312	5.69E-05	0.830
cg23694520	6	30029482	ZNRD1ASP	S_Shore	6114.2	1297.9	-487.2	762.8	6.12E-05	0.830
cg02356111	7	5570277	ACTB	Island	22151.1	4393.1	68.5	2833.9	6.28E-05	0.830
cg08867254	11	6704523	MRPL17	Island	6862.5	1630.4	-539.7	455.6	6.82E-05	0.830
cg23182972	7	22459146	STEAP1B	Sea	3649.9	831.5	-181	448.1	7.66E-05	0.830
Change in HbA1c										
cg24385580	17	28088301	SSH2	Island	158.2	26.9	-30.6	13.8	7.16E-08	0.02
cg17619093	6	31620354	BAG6	Island	521.7	86.6	-71.3	46.9	1.84E-07	0.024
cg06094325	7	157818764	PTPRN2	Sea	44.3	7.4	-16	6.9	2.61E-07	0.024
cg08409451	16	2273123	E4F1	N_Shore	30	5.6	-8.1	3.5	4.66E-07	0.032
cg04015759	2	27718181	FNDC4	Island	45.9	7.8	-4.6	4.6	6.29E-07	0.032
cg09010904	3	51573036	RAD54L2	Island	37.5	6.7	-8.2	4.7	7.02E-07	0.032
cg08942772	19	17651048	FAM129C	Island	21.1	3.8	-2.6	2.5	1.78E-06	0.069
cg06314646	1	226374538	ACBD3	Island	80	16.9	-14.1	8.8	6.12E-06	0.208
cg15285112	17	26215075	NOS2	Sea	24	5	-4.7	3.1	7.46E-06	0.226
cg07872868	2	27530153	TRIM54	Island	17.8	3.4	-3.4	2.7	8.92E-06	0.243
cg21099776	17	17739742	SREBF1	Island	50.9	10.9	-8.1	5.4	9.91E-06	0.246
cg02932026	1	42801455	FOXJ3	S_Shore	118.2	23.8	-24.8	18.5	1.48E-05	0.334
ch.16.54217905R	16	55660404	-	Sea	261.9	56	-24.7	25.2	1.74E-05	0.334
cg07277818	5	137774639	KDM3B	Sea	42	8.7	-5.4	5.3	1.94E-05	0.334
cg12092708	1	19229145	ALDH4A1	Island	133.3	31.2	-25.8	13.7	2.09E-05	0.334
cg10950272	7	42276881	GLI3	Island	116.7	26.1	-14.7	10.2	2.10E-05	0.334
cg01731360	5	49736671	EMB	Island	50.2	10.9	-13.7	7.9	2.18E-05	0.334
cg05278271	19	7554137	PEX11G	S_Shore	143.3	31.6	-25.9	16.7	2.30E-05	0.334
cg25837738	7	148725785	PDIA4	Island	34.9	7.5	-7.7	5.1	2.44E-05	0.334
cg06812693	4	26323246	RBPJ	S_Shore	28.1	5.9	-4.9	3.9	2.64E-05	0.334

Abbreviations: RYGB, Roux-en-Y gastric bypass; BAND,gastric banding; CHR, chromosome; SE, standard error; FDR, false discovery rate; FPG, fasting plasma glucose; HbA1c, hemoglobin A1c.

Supplemental Table S4. CpGs differentially associated with changes in glycemic measures, after Bacon correction

CpG	CHR	Position	Nearest gene	Relation to CpG islands	β (RYGB)	SE(RYGB)	β (BAND)	SE(BAND)	β (IMI)	SE(IMI)	*p* for interaction	FDR for interaction	Bacon.*p* for interaction	Bacon.FDR for interaction
Change in FPG, RYGB vs. IMI														
cg18373318	5	53813164	SNX18	N_Shore	-83.3	70	55	67.5	733.8	111.6	2.26E-09	0.001	7.79E-09	0.002
cg26878734	2	176793622	LNPK	Sea	-481.5	200.6	73.8	121	1244.9	218	3.82E-09	0.001	1.48E-08	0.002
cg05994094	18	31020806	CCDC178	Island	-282	150.3	160.2	199.2	1474.4	253.4	9.61E-09	0.001	4.47E-08	0.004
cg08383526	8	24297818	ADAM7	Sea	656.5	229.9	-195.2	215.3	-1253.1	248.6	1.09E-07	0.007	6.04E-07	0.041
Change in HbA1c, RYGB vs. IMI														
cg18373318	5	53813164	SNX18	N_Shore	-2.2	2.1	1.3	2.1	23.8	3.5	2.36E-09	0.001	1.82E-09	<0.001
cg04340895	4	47463168	COMMD8	N_Shelf	24.8	11.7	-3.2	10.6	-87.8	15	1.16E-07	0.009	2.55E-08	0.003
cg14582478	7	136845265	LOC349160	N_Shelf	83.4	20.3	-0.7	14.3	-59.4	14.4	1.64E-07	0.009	4.01E-08	0.004
cg08336593	16	3280662	ZNF200	N_Shelf	95.4	22.5	12.8	17.9	-62.9	16.4	2.07E-07	0.009	5.42E-08	0.004
cg05081395	11	108345522	KDELC2	Sea	42.1	22.1	16.7	21.7	-148.2	27.4	3.41E-07	0.012	1.03E-07	0.006
cg17619093	6	31620354	BAG6	Island	521.7	86.6	-71.3	46.9	-21.9	41.9	3.98E-07	0.012	1.25E-07	0.006
cg21191176	14	23402052	PRMT5	S_Shelf	34.1	14.8	-16.8	15.6	-97.2	17.2	4.74E-07	0.012	1.56E-07	0.006
cg03863499	3	191670165	-	Sea	-12.7	6	4.4	3.9	36.8	7	6.25E-07	0.014	2.21E-07	0.007
cg26878734	2	176793622	LNPK	Sea	19.7	11.5	1	10.3	-74.4	14	9.37E-08	0.009	2.22E-07	0.007
cg24385580	17	28088301	SSH2	Island	158.2	26.9	-30.6	13.8	-4.3	11.2	8.48E-07	0.017	3.24E-07	0.008
cg01850334	2	101667004	TBC1D8	Sea	33.7	11.7	-2.9	10.4	-58.7	14.1	9.55E-07	0.017	3.76E-07	0.008
cg22964496	10	126718276	CTBP2	S_Shelf	17.8	8.9	-4.4	8.7	-46.7	9.8	9.93E-07	0.017	3.94E-07	0.008
cg23154849	2	209240193	PTH2R	Sea	14.4	5.1	-1.2	5	-19.4	6	1.00E-06	0.017	4.00E-07	0.008
cg06687848	10	118547556	HSPA12A	N_Shore	-12.7	7.1	5.7	7.4	42.3	7.9	1.73E-07	0.009	4.68E-07	0.009
ch.16.54217905R	16	55660404	-	Sea	261.9	56	-24.7	25.2	-74.2	32.2	1.15E-06	0.017	4.73E-07	0.009
cg25549720	6	32058851	TNXB	S_Shelf	32.8	8.6	1.2	7.1	-18.4	6.9	1.35E-06	0.019	5.74E-07	0.009
cg04282607	22	22007286	MIR301B	S_Shore	23.6	4.7	-2.2	4.1	-7	3.8	1.36E-06	0.019	5.80E-07	0.009
cg11971662	8	99499993	STK3	Sea	25.9	12.5	2.4	8.6	-55.7	10.4	1.72E-06	0.02	7.74E-07	0.011
cg13093338	1	192899280	-	Sea	86.1	28.2	-4.3	16.6	-79.4	16	1.73E-06	0.02	7.77E-07	0.011
cg10143823	14	28192481	-	Sea	41.7	12.1	1	10.5	-39.5	10.8	1.76E-06	0.02	7.95E-07	0.011
cg08023442	3	34767621	-	Sea	29.2	12	-5.5	15.4	-73.1	14.5	1.84E-06	0.02	8.42E-07	0.011
cg14580737	19	19301780	BORCS8-MEF2B	N_Shore	16.4	4.8	0.5	4.2	-13.5	5	1.92E-06	0.02	8.85E-07	0.011
cg26646203	4	170925670	MFAP3L	Sea	16	6.6	3.1	8	-33.1	7	2.02E-06	0.02	9.38E-07	0.011
cg04768557	10	62114363	ANK3	Sea	40.4	9.2	2.6	8	-16.7	6.9	2.17E-06	0.021	1.03E-06	0.012
cg24861436	1	223285901	TLR5	Sea	25.6	6.8	-4	6.5	-37.7	10.4	2.26E-06	0.021	1.08E-06	0.012
cg08012844	17	72258169	TTYH2	Sea	16.6	8.6	3.5	9.1	-50.1	10.2	2.82E-06	0.025	1.40E-06	0.014
cg09713515	7	111411453	DOCK4	Sea	20.3	7.1	6.8	8.3	-36.8	9.3	2.97E-06	0.025	1.50E-06	0.014
cg21995919	2	182322279	ITGA4	Island	-467.7	91.5	-62.5	76.1	225.5	92.1	4.73E-07	0.012	1.54E-06	0.014
cg23303246	11	131640135	NTM	Sea	41.4	10.1	1.4	6.1	-16.8	5.3	3.07E-06	0.025	1.56E-06	0.014
cg05994094	18	31020806	CCDC178	Island	-3.2	4.7	6.1	6.5	46.5	8.2	4.84E-07	0.012	1.58E-06	0.014
cg17844553	12	11322611	PRH1-PRR4	N_Shore	97.1	25.2	-8.2	23.4	-80.2	26.7	3.25E-06	0.025	1.67E-06	0.015
cg21044833	19	13010694	GCDH	Sea	26.8	11	-5.5	8	-43.2	10.9	3.53E-06	0.025	1.84E-06	0.016
cg04829143	10	30290657	-	Sea	25.7	12.4	4	16.1	-65.2	13.9	3.65E-06	0.025	1.92E-06	0.016
cg16366449	6	46923611	ADGRF5	Sea	48	16.7	14.3	13.7	-63	15.2	3.69E-06	0.025	1.94E-06	0.016
cg11018488	1	32482522	KHDRBS1	S_Shore	12.1	6.9	-7.9	6.5	-34.5	7.4	4.26E-06	0.026	2.30E-06	0.018
cg06355272	10	3576929	-	Sea	19.8	20	-8	15.8	-126	21.9	4.46E-06	0.026	2.43E-06	0.018
cg16929354	21	34915386	GART	Island	316.3	101.9	-6.3	62.6	-383	106.4	4.67E-06	0.027	2.57E-06	0.019
cg00071565	2	10588493	ODC1	Island	470	139.9	-68.1	135.4	-492.6	136.4	5.35E-06	0.03	3.01E-06	0.022
cg18000650	5	138270099	CTNNA1	Sea	93.6	24	0	22.3	-69.2	26.1	5.81E-06	0.032	3.32E-06	0.023
cg19485804	2	233878714	NGEF	Sea	32.7	17.7	-2.1	10.2	-85.2	16.5	6.03E-06	0.033	3.47E-06	0.024
cg11334822	6	30128686	TRIM10	Sea	19.8	6.9	-4	6.4	-22.3	6.2	6.29E-06	0.033	3.65E-06	0.024
cg08383526	8	24297818	ADAM7	Sea	13.8	7.4	1.2	7	-37.8	8.1	6.79E-06	0.033	3.99E-06	0.025
cg24907075	19	12515481	ZNF799	S_Shelf	28.9	8.3	2	6.2	-22.5	7.6	6.97E-06	0.033	4.12E-06	0.025
cg24766398	6	25830852	SLC17A1	Sea	30.5	8.2	5	6.7	-13.5	5.9	7.00E-06	0.033	4.14E-06	0.025
cg00658652	16	71500215	ZNF23	S_Shelf	7.5	3.8	-2.3	3.5	-16.5	4	7.06E-06	0.033	4.18E-06	0.025
cg09010671	13	79177763	RNF219-AS1	Island	-57.3	29.4	33.1	41.9	194.9	40.2	1.14E-06	0.017	4.25E-06	0.025
cg12627537	22	43485414	TTLL1	Island	21.2	6.4	-4.1	5.2	-32.3	9.4	7.34E-06	0.033	4.37E-06	0.025
cg05789476	6	159065051	DYNLT1	N_Shore	180.5	48.4	-35.4	37.3	-118.4	42.2	7.64E-06	0.033	4.58E-06	0.025
cg03328673	13	22687730	-	Sea	39.2	12.3	-0.8	9	-32.7	9.4	7.65E-06	0.033	4.58E-06	0.025
cg15569451	20	44041324	SYS1-DBNDD2	N_Shelf	31.2	9.8	-13.7	8.5	-31.8	9	7.71E-06	0.033	4.63E-06	0.025
cg08691332	12	72142653	-	Sea	22.4	10.9	1.1	8.3	-43.8	11.8	7.71E-06	0.033	4.63E-06	0.025
cg01554453	6	132129103	ENPP1	Island	14.3	4.1	-7.2	3.3	-12.8	4.4	9.40E-06	0.036	5.83E-06	0.031
cg06168324	2	161349637	RBMS1	Island	-87.7	22.9	-3.3	31.4	102.1	26.5	1.56E-06	0.02	6.04E-06	0.031
cg22503060	12	92368774	-	Sea	166.8	41.5	-1.9	28.9	-75.3	33.3	1.01E-05	0.037	6.36E-06	0.032
cg14784343	11	1293406	TOLLIP	Island	36.8	6.8	4.1	6.2	-7.8	6.3	1.06E-05	0.038	6.73E-06	0.033
cg27568165	1	205631978	SLC45A3	Island	43.1	12.4	3.4	8.4	-20.1	7.8	1.19E-05	0.039	7.66E-06	0.036
cg21393619	13	27829259	RPL21	S_Shelf	68.4	22.6	2.4	16.7	-70	23.5	1.21E-05	0.039	7.84E-06	0.036
cg14038647	3	13396729	NUP210	Sea	41.9	13.6	6.9	11	-42	12.3	1.22E-05	0.039	7.86E-06	0.036
cg16591304	10	115439377	CASP7	Island	115.1	24.7	-6.1	32.2	-46.2	23.2	1.23E-05	0.039	7.95E-06	0.036
cg05844420	2	171621820	-	Sea	93.7	26.6	29.1	18.1	-58.7	21.1	1.23E-05	0.039	7.98E-06	0.036
cg22685442	4	8418050	ACOX3	Sea	45.2	17.7	2.7	18.6	-75	20	1.24E-05	0.039	7.99E-06	0.036
cg01840575	2	38977957	SRSF7	Island	80.6	20.7	-4.6	15.3	-54.4	21.2	1.34E-05	0.041	8.78E-06	0.038
cg10437900	7	23518135	-	S_Shelf	8	3.4	-1.4	4.5	-17.2	4.4	1.34E-05	0.041	8.81E-06	0.038
cg18941458	1	22191585	HSPG2	Island	15	14.7	-7.8	12.6	-73.1	16.3	1.36E-05	0.041	8.91E-06	0.038
cg16508028	8	145678763	CYHR1	S_Shelf	11.3	9.7	0.9	7	-54	10.4	1.39E-05	0.041	9.12E-06	0.038
cg14284174	14	66953088	CCDC196	Sea	34.1	15.2	12.3	12.5	-56.3	13.3	1.50E-05	0.044	1.00E-05	0.041
cg21314288	11	61511042	DAGLA	Island	31.5	10.6	13.6	9	-35.6	9.9	1.54E-05	0.044	1.03E-05	0.042
cg11924517	6	52050597	IL17A	Sea	16.6	8.3	-1.5	6.5	-35.6	8.1	1.55E-05	0.044	1.04E-05	0.042
cg17156402	1	16390468	FAM131C	Sea	25.5	6.6	4.3	5.2	-9.8	5	1.61E-05	0.045	1.09E-05	0.043
cg02935305	6	35995251	SLC26A8	Island	-167.2	57.3	-45.3	48.4	248.3	59.9	2.81E-06	0.025	1.16E-05	0.044
cg12616421	6	32017224	TNXB	Sea	9.4	14.4	6.4	15.3	-110.8	21.2	1.71E-05	0.045	1.16E-05	0.044
cg10134425	4	153934955	-	Sea	38.5	12.2	0.5	9.1	-33.2	11.7	1.71E-05	0.045	1.16E-05	0.044
cg21369919	10	21318882	NEBL	Sea	17.3	12.3	-5.7	10.8	-71	15.4	1.73E-05	0.045	1.18E-05	0.044

Supplemental Table S4. CpGs differentially associated with changes in glycemic measures, after Bacon correction

CpG	CHR	Position	Nearest gene	Relation to CpG islands	β (RYGB)	SE(RYGB)	β (BAND)	SE(BAND)	β (IMI)	SE(IMI)	*p* for interaction	FDR for interaction	Bacon.*p* for interaction	Bacon.FDR for interaction
cg00459898	2	241912309	-	Sea	44.5	14.4	6.5	11.3	-40.8	13.7	1.77E-05	0.045	1.21E-05	0.044
cg21834048	3	141495562	GRK7	N_Shore	142.5	35.6	10.3	24.8	-48.1	23.8	1.78E-05	0.045	1.22E-05	0.044
cg13057898	1	3703894	LRRC47	Island	6.5	3.1	0.4	2.2	-10.2	2.6	1.80E-05	0.045	1.23E-05	0.044
cg25390432	19	45003865	ZNF180	N_Shore	20.3	3.5	1.2	3.1	-0.8	2.9	1.81E-05	0.045	1.24E-05	0.044
cg11424665	3	129158811	MBD4	Island	16.3	5.3	-3.3	3.8	-18.8	6.4	1.88E-05	0.046	1.30E-05	0.045
cg01750895	17	17463041	PEMT	N_Shelf	28.6	9	4.8	4.9	-20.1	6.6	1.94E-05	0.047	1.34E-05	0.046
cg09267427	12	124953924	NCOR2	S_Shelf	-15.4	3.6	0.4	4.3	11.4	4.5	3.30E-06	0.025	1.38E-05	0.047
cg17037611	11	36023494	LDLRAD3	Sea	36.5	16	14.4	10.9	-51	12.8	2.04E-05	0.049	1.42E-05	0.047
cg01294490	6	35656906	FKBP5	S_Shore	-25	8.3	12.5	6.7	60.7	14.8	3.41E-06	0.025	1.43E-05	0.047
cg14441262	6	33140769	COL11A2	Sea	16.2	6.3	0.2	6.1	-17.7	5.3	2.06E-05	0.049	1.44E-05	0.047
cg05131202	15	79352412	RASGRF1	Sea	23.8	12.4	-7.3	12.3	-63.8	15.2	2.14E-05	0.05	1.50E-05	0.049
Change in HbA1c, RYGB vs. BAND														
cg24385580	17	28088301	SSH2	Island	158.2	26.9	-30.6	13.8	-4.3	11.2	7.16E-08	0.02	2.35E-09	0.001
cg17619093	6	31620354	BAG6	Island	521.7	86.6	-71.3	46.9	-21.9	41.9	1.84E-07	0.024	1.01E-08	0.001
cg06094325	7	157818764	PTPRN2	Sea	44.3	7.4	-16	6.9	7.5	8.7	2.61E-07	0.024	1.72E-08	0.002
cg08409451	16	2273123	E4F1	N_Shore	30	5.6	-8.1	3.5	-6.1	6.1	4.66E-07	0.032	4.08E-08	0.003
cg04015759	2	27718181	FNDC4	Island	45.9	7.8	-4.6	4.6	-0.7	7.5	6.29E-07	0.032	6.36E-08	0.003
cg09010904	3	51573036	RAD54L2	Island	37.5	6.7	-8.2	4.7	0.7	7.8	7.02E-07	0.032	7.46E-08	0.003

Abbreviations: RYGB, Roux-en-Y gastric bypass; BAND, gastric banding; IMI, intensive medical intervention; CHR, chromosome; FDR, false discovery rate; FPG, fasting plasma glucose; HbA1c, hemoglobin A1c.

Supplemental Table S5. Top 20 CpGs differentially associated with changes in glycemic measures, RYGB vs. IMI (sensitivity analys

CpG	CHR	Position	Nearest gene	Relation to CpG islands	β (RYGB)	SE(RYGB)	β (IMI)	SE(IMI)	*p* for interaction	FDR for interaction
Change in FPG										
cg18373318	5	53813164	SNX18	N_Shore	-55.3	72.3	722.8	111.1	1.56E-08	0.003
cg26878734	2	176793622	LNPK	Sea	-432.7	201.9	1211.9	217.4	2.30E-08	0.003
cg05994094	18	31020806	CCDC178	Island	-261.6	149.9	1425.8	254.1	3.69E-08	0.003
cg19512521	14	23821902	SLC22A17	Island	-4152.3	1942.4	10071.8	1777.9	2.36E-07	0.016
cg21191176	14	23402052	PRMT5	S_Shelf	796.1	455	-3389.6	529.9	3.69E-07	0.018
cg08383526	8	24297818	ADAM7	Sea	570.9	233.8	-1245.9	245.8	3.99E-07	0.018
cg18941458	1	22191585	HSPG2	Island	882.7	457.2	-2183.7	495.7	5.65E-07	0.018
cg01139906	3	10290027	IRAK2	Island	-730.8	343.4	2404.9	473.9	6.28E-07	0.018
cg03467087	6	158448134	SYNJ2	Sea	-334.7	117.1	544.6	128.4	6.68E-07	0.018
cg03831971	4	100868132	DNAJB14	Island	-1674	521.1	3379.5	769.1	6.77E-07	0.018
cg10069716	16	30922445	-	Sea	990.2	292.9	-792.6	255.3	8.49E-07	0.019
cg04340895	4	47463168	COMMD8	N_Shelf	748.4	385.5	-2750.1	490.8	8.98E-07	0.019
cg08691332	12	72142653	-	Sea	776.4	336.7	-1478.1	360.4	9.67E-07	0.019
cg00658652	16	71500215	ZNF23	S_Shelf	235.5	114.7	-579.8	123.9	9.71E-07	0.019
cg23724489	6	92401486	-	Sea	815.8	193.5	-397	158.7	1.15E-06	0.021
cg15087376	3	149113539	-	Sea	-2308.6	564.9	1669.4	524.4	1.28E-06	0.021
cg27431274	12	107979028	BTBD11	S_Shelf	598.3	159.5	-409.7	157.4	1.32E-06	0.021
cg12606891	1	7913872	UTS2	Sea	588.8	206.3	-765	205.5	1.38E-06	0.021
cg26232102	1	155024228	DCST1	Island	-2566.5	812.3	2934.4	685.9	1.51E-06	0.022
cg12870750	17	7791882	CHD3	S_Shelf	-90.4	81.3	599.2	113.5	1.64E-06	0.022
Change in HbA1c										
cg18373318	5	53813164	SNX18	N_Shore	-1.3	2.2	23.6	3.5	9.57E-09	0.001
cg04340895	4	47463168	COMMD8	N_Shelf	31.2	11.3	-87	14.2	1.09E-08	0.001
cg14582478	7	136845265	LOC349160	N_Shelf	73.7	19.1	-72.4	14	1.76E-08	0.002
cg17619093	6	31620354	BAG6	Island	512.5	84.1	-31	40.8	2.15E-07	0.015
cg21995919	2	182322279	ITGA4	Island	-477.3	88.4	208	89.2	2.93E-07	0.015
cg08336593	16	3280662	ZNF200	N_Shelf	91.5	22.3	-61.6	16.1	3.58E-07	0.015
cg26878734	2	176793622	LNPK	Sea	-11.1	6	35.6	6.9	3.98E-07	0.015
cg06687848	10	118547556	HSPA12A	N_Shore	-12.7	7.1	40.4	7.9	4.50E-07	0.015
cg05081395	11	108345522	KDELC2	Sea	38.4	21.9	-144.6	27	6.81E-07	0.021
cg21191176	14	23402052	PRMT5	S_Shelf	34.1	14.6	-93.5	17.2	7.68E-07	0.021
cg08023442	3	34767621	-	Sea	32	11.8	-70.8	14.1	1.03E-06	0.026
cg05994094	18	31020806	CCDC178	Island	-2.7	4.7	45	8.2	1.18E-06	0.027
cg23154849	2	209240193	PTH2R	Sea	14	5	-18.9	5.9	1.43E-06	0.03
cg06695833	19	18220682	MAST3	N_Shore	129.4	33.1	-121.1	36.1	1.61E-06	0.031
cg03863499	3	191670165	-	Sea	17.3	11.5	-72.4	13.9	1.91E-06	0.031
cg17844553	12	11322611	PRH1-PRR4	N_Shore	98.7	24.5	-77.9	25.9	1.98E-06	0.031
cg24861436	1	223285901	TLR5	Sea	26.2	6.6	-35.7	10.2	2.30E-06	0.031
cg24385580	17	28088301	SSH2	Island	151.8	26.6	-1.7	11.1	2.36E-06	0.031
cg26646203	4	170925670	MFAP3L	Sea	14.6	6.5	-33.1	6.9	2.43E-06	0.031
cg09010671	13	79177763	RNF219-AS1	Island	-53.5	29.3	189.2	40	2.54E-06	0.031

Abbreviations: RYGB, Roux-en-Y gastric bypass; IMI, intensive medical intervention; CHR, chromosome; SE, standard error; FDR, false discovery rate; FPG, fasting plasma glucose; HbA1c, hemoglobin A1c. Models were additionally adjusted for use of hypoglycemic drugs.
